# Medicinal and combined medicinal/recreational cannabis use in California following the passage of Proposition 64

**DOI:** 10.1186/s42238-025-00285-9

**Published:** 2025-07-12

**Authors:** Daniel Ageze, Renee Dell’Acqua, Thomas D. Marcotte, Jill Rybar, Sara Baird, Alice Gold, Tom Shaughnessy, Ilene Lanin-Kettering, Linda Hill

**Affiliations:** 1 Herbert Wertheim School of Public Health and Human Longevity Science, UC San Diego, 9500 Gilman Drive, La Jolla, CA 92093-0994 USA; 2 Department of Psychiatry, Center for Medicinal Cannabis Research, UC San Diego, 220 Dickinson Street, Suite B, MC8231, San Diego, CA 92103-8231 USA; 3 Quester, 6500 University Avenue, Suite 205, Des Moines, IA 50324 USA

**Keywords:** Cannabis, Medicinal use, Recreational use, Pain, Patient-provider relationship, Side effects, Dispensaries

## Abstract

**Background:**

Proposition 64, the Adult Use of Marijuana Act of 2016, reshaped cannabis use in California. This study explores the use patterns of people who use cannabis for medicinal-only and combined medicinal and recreational use after implementation of Proposition 64.

**Methods:**

A quantitative, population-based online questionnaire included 4,020 current cannabis users, 523 former users, and 635 non-users. This analysis focuses on participants who self-identified as using cannabis for medicinal-only (*n* = 711) or both medicinal and recreational (M + R, *n* = 1719) purposes.

**Results:**

Sixty one percent of current cannabis users report medicinal use. Medicinal-only users were more likely to be female (OR 1.6, *p* < 0.001), have kids in household (OR 1.5, *p* < 0.001), and began cannabis use later (mean age 34 vs. 23, *p* < 0.001). Pain relief was the predominant reason for use, followed by sleep, anxiety, and stress relief. While both groups reported positive effects, M + R users experienced more negative side effects. Fewer medicinal-only users cited a desire to “feel the high,” (42% vs. 75% M + R, *p* < 0.001). Medicinal-only users felt less comfortable discussing cannabis with primary care providers than M + R users (75% vs 83%, *p* < 0.01). All users were more likely to seek information online (44–57%) or from friends/family (47–52%) than health professionals (26–27%). Dispensaries were the main cannabis source for both medicinal groups (72% M vs. 84% M + R, *p* < 0.01), with licensure being very or extremely important (72% M, 66% M + R, *p* < 0.01). Monthly spending for medicinal-only users was lower ($127 vs. $186 for M + R, *p* < 0.001), and they were more likely than M + R users to wait before feeling safe to drive after using cannabis.

**Conclusion:**

People who use medicinal cannabis alone vary in key areas from people who use cannabis for both medicinal and recreational reasons. The need for better patient-provider relationships and clinically informed guidance is evident to support medicinal cannabis users.

## Background

Cannabis is a complex plant containing 540 cannabinoids that has a long history of therapeutic applications in various continents including Europe, North America, and the rest of the world (National Center for Complementary and Integrative Health 2025). While strict regulations and use prohibitions became widespread in the United States in the 1920 s and 1930 s with the passing of laws such as the Uniform Narcotic Drug Act and the Marihuana Tax Act of 1937, there has been a shift in legislation over the last three decades towards the legalized use of cannabis (National Conference of State Legislatures 2022). In 1996, California became the first state to allow medicinal cannabis use through Proposition 215: The Compassionate Use Act. As of April 2023, 38 states, 3 territories, and the District of Columbia permit the use of cannabis products for therapeutic purposes, and 24 for recreational use (National Conference of State Legislatures 2022). At the federal level, however, cannabis with delta-9-tetrahydrocannabinol (THC) (excluding hemp products with < 0.3% THC on a dry weight basis) remains a Schedule I drug under the Controlled Substances Act, classified as a substance with “no currently accepted medical use in the United States, a lack of accepted safety for use under medical supervisions, and high potential for abuse” (Drug Enforcement Administration 2025).

Along with legalization, research around the medicinal use of cannabis has expanded. A 2017 review from the National Academy of Sciences concluded that cannabis may have benefits for certain conditions (e.g., neuropathic pain) (Drug Enforcement Administration 2020; National Academies of Sciences, Engineering, and Medicine 2017). The Food and Drug Administration (FDA) has approved the use of pharmaceutical-grade cannabinoids, such as synthetic THC (dronabinol, nabilone) and plant-derived cannabidiol (Epidiolex) to treat a select medical conditions including nausea and vomiting caused by chemotherapy, loss of appetite and weight loss in HIV, and symptoms associated with certain seizure disorders (U.S. Food and Drug Administration 2025). Broader indications for use of cannabis to treat chronic pain, muscle spasticity, multiple sclerosis, irritable bowel syndrome, and mental health disorders are currently being explored, with some positive but inconsistent findings in some areas (Klimkiewicz and Jasinska 2017). In response to increasing data supporting its use, the DEA is considering a proposal to reschedule cannabis to schedule III (Moritz College of Law 2025). Yet several medical societies, including the American Medical Association, American Society for Addiction Medicine, and the American College of Obstetricians and Gynecologists, have maintained a cautious stance, stating that there is insufficient evidence for its medicinal use (American Medical Association. 2025; American Society of Addiction Medicine 2020; American College of Obstetricians and Gynecologists 2017). Despite this lack of guidance, the use of cannabis for medical reasons continues to grow. In 2018, it was estimated that 27% of people who use cannabis nationwide and 34% in cannabis legal states used cannabis for medicinal reasons (Leung et al. 2022). This is a stark increase from one 2012 study that estimated that 5% of California adults had ever used medicinal cannabis, though this may be an underestimate as it occurred when cannabis use less acceptable (Ryan-Ibarra et al. 2015).

In 2016, Proposition 64: The Adult Use of Marijuana Act (Prop 64), legalized recreational cannabis in California, altering the landscape for patients who use medicinal cannabis (Drug Enforcement Administration 2025). Following implementation of Prop 64 and the establishment of regulated dispensaries, there was a dramatic increase in the availability of various cannabis products, including different dosages and formulations (Padon et al. 2022). People seeking to use medicinal cannabis no longer needed a physician recommendation, expanding access while potentially impacting patient-provider communication (Ryan et al. 2021; California Department of Public Health n.d.). A 2021 systematic review suggests that reducing prescription medication is a motivating factor for medicinal cannabis use (Zeng et al. 2021). A 2019 survey found that people who use cannabis for medicinal and recreational purposes are more likely to report use of inhaled products than people who use cannabis for medicinal-only reasons, and are less likely to consult with a healthcare professional (Boehnke et al. 2019), a beginning into identifying differences between these two medicinal use groups. However, although these and other studies have begun exploring the characteristics of medicinal cannabis users in this new climate, these have been often limited by evaluating only select populations (e.g. individuals with chronic pain). A comprehensive understanding of factors about how medicinal cannabis is used in a post-legalization landscape will highlight key factors for future public health interventions, inform medical providers, and guide future research into this evolving field.

The purpose of this study was to explore the patterns of THC-containing cannabis use for medicinal-only and combined medicinal-recreational purposes in a population-matched sample of California adults since implementation of Prop 64, including behaviors, symptoms, perceptions of patient-provider relationships, sources of information, and positive and negative effects of use.

## Methods

Impact 64, funded by the State of California Department of Cannabis Control, used a comprehensive, three-tiered mixed-methods design to study cannabis use patterns in CA. This included: 1) semi-structured qualitative interviews with 23 Subject Matter Experts (SMEs); 2) a preliminary questionnaire with 200 participants; and 3) a detailed questionnaire of 5,000 California adults, with a goal of 4,000 current cannabis users, 500 former users, and 500 non-users. This analysis focuses on a subset of results from the final phase. The University of California San Diego Institutional Review Board (IRB) granted approval for all research activities.

### Questionnaire development

The questionnaire was designed to take up to 25 min, and was designed and implemented in partnership with Quester, a market analysis firm. Questionnaire content was informed by the SME interviews to incorporate key domains (use type, source, knowledge, reason for use, attitude, consequences) and refined based on results of the preliminary questionnaire. In this study, cannabis was defined as products containing THC, as explicitly specified in the questionnaire.

### Participants and recruitment

Through quota sampling, participants completed a brief screening questionnaire that anonymously collected demographic and cannabis usage data. Participants were unaware of the purpose of the study at the time of the screening questionnaire. Recruitment was designed to align the study participant demographics with the demographic makeup of California's 2020 census. Eligibility criteria included being a California resident, over 21 years old, and fluent in English or Spanish. Those working in the cannabis industry, market research, or public relations were excluded.

From this initial pool, a subset of participants was selected to complete the detailed questionnaire, aiming for a total of 5,000 participants segmented into three groups: people who currently use cannabis (“current users”; who had used within the past three months; *n* = 4,000), people who formerly used cannabis (“former users”; who had not used in at least four months; *n* = 500), and people who have never used cannabis (“non-users”; *n* = 500). Selection for the full questionnaire was based on demographic and cannabis use history, with goal demographics defined by the demographic makeup of each subgroup of the screener questionnaire. The full questionnaire was administered immediately following the initial screening questionnaire, with recruitment ending once the desired sample size was reached.

### Data collection

The questionnaire was launched on December 2, 2022, and closed on February 6, 2023, after meeting recruitment goals. Participants completed the questionnaire online, which was compatible with both desktop and mobile devices.

### Data analysis

Using iterative proportional fitting (rake weighting), demographic weighting was applied to the results of the initial screener questionnaire to match California's census demographics across four key variables: age, gender, race/ethnicity, and income. These initial screener results were used to establish the target demographic profiles for each subgroup of cannabis users (current, former, and non-users).

Each subgroup within the full questionnaire was then weighted to reflect the specific demographic targets of their subgroup. This allowed the screener data to match California’s broader demographics, while the demographics of each full questionnaire subgroup mirrors the specific demographics of that user category within the state.

Statistical analyses, including descriptive statistics, chi-square tests, and multinomial logistic regression, were utilized to examine differences between groups, controlling for demographic factors. Statistical processing was conducted using SPSS v. 28.0.0.0 and JMP Pro v. 17.0.0.22 (JMP 2021; IBM 2022). A significant threshold was set at *p* < 0.05.

## Results

A total of 15,309 individuals completed the initial ‘screener’ questionnaire with demographics and 15,200 provided both demographics and cannabis use information. See Appendix 1 and Appendix 2 for a summary of unweighted and weighted/target screener demographics.

Of the 15,200 initial participants who reported cannabis use history, 37% reported current cannabis use, 30% were former users, and 33% were cannabis non-users.

A total of 5,178 individuals completed the full questionnaire; this included 4,020 current users, 523 former users, and 635 non-users. The remaining 10,022 participants either did not qualify (*N* = 7,367, e.g. individuals who use cannabis less than once every 3 months), were not selected due to quotas (*N* = 991) or were excluded for incomplete participation (*N* = 1,664). After weighing the subset populations to match the demographics of their respective use groups, there was 0% difference from target key demographics. Weighted percentages are presented below unless otherwise specified.

Of the 4,020 current users, 2,430 participants (unweighted, 61%) reported using cannabis for medicinal purposes and were included in this analysis; this included 711 (18% of current users) who reported using cannabis for medicinal purposes only (“medicinal-only”), and 1719 (43% of current users) who reported both medicinal and recreational (M+R) use. The remaining 39% reported recreational use only and were excluded from analysis.

### Demographics

Demographics of the participants who used medicinal-only and M + R cannabis can be found in Table 1. After adjusting for other variables, medicinal-only users were more likely than M + R users to be female (OR 1.6, *p* < 0.001), started cannabis use at an age older than 24 (OR 3.1–9.1, *p* < 0.001), and were more likely to have kids in the household (OR 1.5, *p* < 0.001). Mean monthly spending on cannabis was significantly higher among the M + R group $186 (SD 235) than medicinal-only users $127 (SD 176, *p* < 0.001) after bivariate analysis. 33–34% of participants in both groups had Medical Marijuana Identification Card (MMIC); past cardholders were more likely to be from the M + R group and those that never had one from the medicinal-only group.

**Table 1:** Demographics among medicinal only users and M + R group

Demographics	Medicinal-only (M) *N* = 711	Medicinal & recreational (M + R) *N* = 1719	Adjusted Odds Ratio (*P*-value)^&^
**Gender**			
Male (ref)	48%	62%**	
Female	52% **	38%	OR = 1.6 (< 0.001)
**Age**			
Mean	46 (SD 15) **	42 (SD 13)	NS
21—25 (ref)	9%	8%	
26—35	22%	33% **	
36—45	20%	23%	
46—55	17%	18%	
56—65	19% **	12%	
Over 66	13% **	6%	
**Ethnicity/Race**			
White non-Hispanic (ref)	40%	40%	NS
Hispanic	41%	40%	
Black non-Hispanic	6%	8%	
Asian/Pacific Islander	10%	9%	
**Educational status**			
High school diploma or lower	18%	15%	NS
Some college or college degree (ref)	69%	71%	
Graduate degree	14%	14%	
**Annual Household Income**			
Under 50 K	24%	25%	NS
50–100 K (ref)	28%	28%	
Greater than 100 k	48%	47%	
**Employment status**			
Employed full-time (ref)	53%	64%**	NS
Employed part-time	14%	12%	
Unemployed	32%	24%**	
**Marital Status**			
Single	41%	44%	NS
Married or has partner (ref)	59%	56%	
**Kids in household**			
No kids	53%**	59%	OR = 1.5 (< 0.001)
Have kids (ref)	47%**	41%	
**Region of California**			
Northern region	24%	26%	NS
Central region	17%	16%	
Southern region (ref)	59%	58%	
**Age of first use**			
Mean age	34 (SD 18)**	23 (SD 11)	
Younger than 17 (ref)	18%**	37%	
18–24	25%	35%**	OR = 1.7 (< 0.001)
25–34	18%	14%	OR = 3.1 (< 0.001)
35–44	10%	7%	OR = 3.0, (< 0.001)
45 +	29%**	7%	OR = 9.1, (< 0.001)
**Use frequency**	Medicinal-only (M)	Medicinal & recreational (M + R)	These variables were not included in the multinomial regression analysis
Occasional use	39%**	21%	
Frequent use	36%	32%	
Very frequent use	25%	47%**	
**Mean monthly cost (dollars)**	127 (SD 176)	186 (SD 235) **	
**Medicinal marijuana identification cardholder status**	Medicinal use only (*N* = 711)	Medicinal use & recreational use (M + R group; *N* = 1719)	
Current holder	33%	34%	
Past cardholder	28%	37%**	
Never had one	38%**	29%	

^&^Odds of being Med only users after adjusting for demographics via multi-nominal regression (only OR with statistical significance are shown)

^**^Significance level of < 0.001 after bivariate Chi-square analysis

*NS* Not significant

### Characteristics of cannabis use

The frequency and product types used by medicinal-only and M + R cannabis users are summarized in Table 2. Medicinal-only users were less likely to report being a very frequent cannabis user (multiple times per day) than M + R users (25% vs 47%, *p* < 0.001). The M + R group user group used nearly all cannabis product types at a significantly higher percentage than medicinal-only users, with only topical and oil/tinctures showing no significant difference.

**Table 2:** Characteristics of use

**Frequency of cannabis use**	**Reasons for use** **Weighted %**	**Percent use^**	**Demographical analysis~ & Odd ratio (comparing stated use frequency vs other use frequencies)**
Very frequent user (Multiple times a day)	**M (*N*** **= 711)**	25%	GED (vs graduate degree, OR=1.8*), started cannabis use younger <17 (OR for 45+=5.2**)
	**M+R (*N*** **= 1719)**	47%**	Male (OR=1.4**), not Asian/Pacific islander (OR=0.4–0.5**), HH income <50k (vs 100k+, OR=2.2**), started cannabis use younger <17 (OR for 45+=10.0**)
Occasional user (1–3 times a week or less)	**M**	39%**	Female (OR=1.6**), College & some college (vs GED, OR=2.2**), started cannabis use older age (OR for 45+=2.1**)
	**M+R**	21%	Female (OR=1.4**), HH income 100+ (vs <50k, OR=1.8**), started cannabis older age (OR for 45=3.3**)
**Product type**	**Medicinal use only (*N*** **= 711)**	**Medicinal & recreational use (M+R group;** ***N*** **= 1719)**	
Dried flower	34%	65%**	
Edibles	48%	53%*	
Vaping	22%	43%**	
Topical/transdermal	28%	24%	
Oil/tinctures	18%	20%	
Dabbing	11%	24%**	
Beverages	11%	18%**	
Other forms (Delta 8, 10, V)	4%	8%**	

*Statistically significant *p*-value < 0.05

**p-value <0.001, using Chi-square analysis

^percent use compared M vs M+R using chi-square analysis

~Multinomial regression analysis

Use frequency by medicinal user type was evaluated after adjusting for demographic variables (Table 2). Among medicinal-only users, those who used very frequently were more likely than frequent and occasional users to have a high school degree or lower (vs graduate degree, AOR 1.8, *p* < 0.001), and started cannabis at 17 years or younger (vs. 45 +, AOR 5.2, *p* < 0.001). Among the M + R group, very frequent users were more likely to be male (AOR 1.4, *p* < 0.001), ethnicities other than Asian/Pacific Islander (AOR 0.4–0.5, *p* < 0.001), have a household income less than $50 k (vs > 100 k, AOR 2.2, *p* < 0.001), and started cannabis use at age 17 or younger (vs 45 +, AOR 10.0, *p* < 0.001).

### Reasons for cannabis use

The medicinal-only group was significantly less likely than the M + R group to use cannabis for stress relief (49% vs 75%), anxiety (53% vs 72%), sleep issues (57% vs 69%), PTSD (18% vs 24%), and decreased appetite (10% vs 24%) (*p* < 0.001 for all; Table 3). Overall, however, medicinal-only users were more likely than M + R users to report using cannabis for mental health symptoms (88% vs 62%, *p* < 0.001), while the M + R group were more likely than medicinal-only users to use cannabis for physical symptoms (88% vs 84%, *p* < 0.001). Members of the M + R group were more likely to use cannabis to alleviate 2 or more symptoms and significantly more inclined to desire the high from cannabis than medicinal-only users (75% vs 42%, *p* < 0.001, Table 4). Multivariate analysis by demographics is shown in Table 3.

**Table 3:** Reasons for use of cannabis

Symptoms	Reasons for use	Percent use^	Demographical analysis ~ & Odd ratio (comparing stated reason for use vs not stated reason)	
Stress	**M (*N*** **= 711)**	49%	HH income under 50 k (vs 100 k +, OR = 1.7*), use decreased with age**	
	**M + R (*N*** **= 1719)**	75%**	Female (OR = 1.3*), white non-Hispanic (vs Black, OR = 3.0**), decreased with age**	
Anxiety	**M**	53%	Female (OR = 1.6**), Asian/Pacific islander (vs Hispanic, OR = 2.2**), HH income under 50 k (vs 100 k, OR = 2.7**), use decreased with age **	
	**M + R**	72%**	Female (OR = 1.4**), HH income under 50 k (OR = 1.5**), decreased with age**	
Sleep problems	**M**	57%	Female (OR = 1.6**), Northern CA region (vs Central, OR = 1.8*), started cannabis younger age than 17 (OR for 45 + = 2.1**)	
	**M + R**	69%**	Female (OR = 1.4**), single (OR = 1.3*), Northern CA region (vs Central, OR = 1.4*), decreased with age**	
Pain	**M**	62%	Female (OR = 1.6**), use increased with age**	
	**M + R**	64%	Unemployed (vs employed full-time, OR = 1.6**), started cannabis younger age than 17 (OR for 25 to 34 = 2.1**), increased with age	
PTSD	**M**	18%	Have kids (vs no kids, OR = 2.3**), HH income under 50 k (vs 100 k, OR = 2.2**), started cannabis younger than 17 (vs OR for 45 + = 4.0**)	
	**M + R**	24%**	Female (OR = 1.3*), have graduate degree (vs GED, OR = 2.4**), HH income under 50 k (OR = 2.1**), started cannabis younger than 17 (vs OR for 25–34 = 1.5**)	
Decreased appetite	**M**	10%	Started cannabis younger than 17 (vs OR for 45 + = 10.3**)	
	**M + R**	24%**	HH under 50 k (OR = 1.5*), started cannabis younger than 17 (OR for 45 + = 2.7**), use decreased with age**	
	**Medicinal use only (*N*** **= 711)**	**M + R group (*N*** **= 1719)**		***P*-value**
Any Physical symptom^+^	85%	89%**		< 0.001
Any Mental symptom^+^	62%	88%**		< 0.001
4 symptoms^+^	18%	32%**		< 0.001
3 symptoms^+^	20%	31%**		< 0.001
2 symptoms^+^	32%	23%**		< 0.001

^*^Statistically significant *p*-value < 0.05

**p-value < 0.001

^percent use compared M vs M + R using chi-square analysis

~Multinomial regression analysis

^+^Physical health (sleep, pain, appetite) and mental health (anxiety, PTSD, stress) variables were created by combining the symptoms listed

**Table 4:** Factors impacting the provider-patient relationship and cannabis use

	Medicinal use only (*N* = 711)	M + R group (*N* = 1719)	*P*-value
**Primary doctor is accepting/in favor of medicinal cannabis use**	66%	68%	0.3
**Primary doctor is accepting/in favor of recreational cannabis use**	44%	56%	0.0001
**Felt comfortable talking to primary doctor about cannabis**	75%	83%**	0.0001
**Primary doctor is aware of medicinal cannabis use**	66%	73%**	0.0001
**Provider informed them of possible drug interaction**	63%	66%*	0.03
**Use cannabis instead of prescribed medications**	59%	61%	0.3
Desire to feel the high in addition to symptomatic relief	42%	75%**	0.0001
Symptoms addressed with cannabis	**Cannabis use instead of prescribed medications (*N*** **= 1452)**	**Cannabis use in addition to prescribed medications (*N*** **= 961)**	***P*-value**
**Stress**	63%	37%	0.7
**Anxiety**	62%	38%	0.05
**Sleep problems**	65%	35%*	0.03
**Pain**	59%	41%**	0.0001
**PTSD**	58%	42%**	0.005
**Decreased appetite**	65%	35%	0.17
Reasons not to feel comfortable discussing cannabis with primary doctor	**Medicinal use only (Out of 20%)**	**M + R group (Out of 13%)**	***P*-value**
**Fear of judgment**	48%	57%	0.05
**Fear of stigma**	36%	47%*	0.02
**Do not feel they understand**	19%	21%	0.5
**Do not feel provider is knowledgeable about cannabis**	13%	13%	0.8
**Negative past experience**	48%	57%	0.1

^*^Statistically significant *p*-value < 0.05

**p-value < 0.001

^percent use compared M vs M + R using chi-square analysis

~Multinomial regression analysis

### Patient-provider relationship

Participants in both groups believed their primary doctors were favorable towards medicinal cannabis use (66–68%, see Table 4). Fewer medicinal-only users felt their doctors accepted recreational cannabis use (44% vs. 56%, *p* < 0.001). A lower percentage of the medicinal-only group (75%) felt comfortable discussing cannabis with their healthcare providers compared to the M + R group (83%, *p* < 0.001). Additionally, more M + R group members reported that their primary doctor knew of their medicinal cannabis use (73% vs 66%, *p* = 0.0001). Those uncomfortable discussing cannabis with their doctors mainly cited fear of stigma. About 60% of participants in both groups used cannabis instead of prescription medications, which was not statistically significant. Among those using it for pain, PTSD, and sleep problems, they were significantly more likely to use cannabis instead of prescribed medications.

### Sources of information

The internet and family/friends were the primary sources of cannabis-related information for both use groups (Table 5). The M + R group more frequently cited the internet, family/friends, budtenders, and workplace sources. About a quarter (26–27%) of each group consulted health professionals (e.g., doctors, nurse practitioners, therapists, or non-doctor clinicians like naturopaths or nutritionists) for information. As it relates to MMIC, of the 829 MMIC holders, 65% got their recommendations from primary care doctors, while 34% used alternative providers, including online sources. Among internet users, medicinal-only users were more likely to be Asian/Pacific Islander, while M + R users often held graduate degrees. Users relying on family/friends in the M + R group were predominantly male, typically had high school or lower education, and were often Hispanic or Black non-Hispanic with children at home. Those seeking advice from budtender generally started using cannabis at a young age and were female in both groups while those in the M + R group members likely had college education and had no children at home. Those seeking out health professionals in M + R group were likely single, had household income of less than $50k, unemployed, and lived in central CA.

**Table 5:** Source of information among medicinal and M + R cannabis users

**Source of information**	**Reasons for use**	**Percent use^**	**Demographical analysis ~ & Odd ratio (comparing stated source of information vs not stated source)**
Internet	**M (*N*** **= 711)**	44%	Asian/Pacific islander (OR = 2.1–2.5**)
	**M + R (*N*** **= 1719)**	57%**	Have graduate degree (OR = 2.1**), no kids in HH (vs have kids, OR = 1.3*)
Family/Friends	**M**	47%	Decreased with age**
	**M + R**	52%**	Male (OR = 1.4**), GED or College (vs professional degree, OR = 1.7**), have kids in HH (vs no kids, OR = 1.5**)
Budtenders	**M**	28%	Female (OR = 1.7**), started cannabis younger than 17 (vs OR for 45 + = 2.1**)
	**M + R**	42%**	Female (OR = 1.3**), college degree (vs graduate, OR = 1.7**), no kids in HH (vs no kids, OR = 1.3*), started cannabis younger than 17 (OR for 25 to 34 = 1.9**), use increased with age**
Health professional (Physicians/therapist)	**M**	27%	Use increased with age**
	**M + R**	26%	Single (OR = 1.3*), HH income under 50 k (vs 100 k, OR = 1.6**), unemployed (vs employed full-time, OR = 1.8**), no kids in HH (OR = 1.5*), lived central CA region (vs southern, OR = 1.5*), increased with age**
Workplace	**M**	7%	Male (OR = 2.0*), have kids in HH (vs no kids, OR = 2.2**)
	**M + R**	10%*	Male (OR = 2.0**), married (OR = 1.7**), have kids in HH (vs no kids, OR = 1.6**), lived Southern CA region (vs Central, OR = 2.1**), decreased with age**
**Source of cannabis**	**Reasons for use**	**Percent use^**	**Demographical analysis & Odd ratio (comparing stated source of cannabis vs not stated source)**
Dispensary	**M**	72%	Female (OR = 1.8**), employed part-time (vs unemployed, OR = 2.3**),
	**M + R group**	84%**	Single (OR = 1.4*), decreased with age*
Delivery	**M**	28%	Married (OR = 1.5*), have kids in HH (vs no kids, OR = 1.6**), started cannabis younger than 17 (vs OR for 45 + = 2.0*)
	**M + R**	45%**	Have graduate degree (vs GED, OR = 2.1**), employed full-time (vs unemployed, OR = 1.8**), have kids in HH (vs no kids, OR = 1.4**), decreased with age**
Family/Friends	**M**	22%	NS
	**M + R**	36%**	HH under 50 k (OR = 1.5**), lived in Northern CA than southern (OR = 1.4**), started cannabis younger than 17 (OR for 45 + = 3.3**)
Grow their own	**M**	10%	Had kids in HH (vs no kids, OR = 1.8*), married (OR = 2.0*), started cannabis younger than 17 (vs OR for 45 + = 3.7**)
	**M + R**	14%*	Male (OR = 2.2**), white non-Hispanic (vs Black non-Hispanic, OR = 3.5**), married (OR = 1.5**), lived in Northern CA (vs southern, OR = 1.9**), started cannabis older than 17 (OR for 45 + = 2.4**)
How important is it to you that the dispensary/delivery service is licensed	Reasons for use	Percent use^	Demographical analysis & Odd ratio (vs somewhat or not important)
Very or extremely important	M	72%**	Have graduate degree (OR = 3.6**), started cannabis older than 17 (OR = 3.0**)
	M + R	66%	Graduate degree (vs college, OR = 1.5*), HH income 100 k + (vs under 50 k, OR = 1.6**), Northern CA region (vs central OR = 1.5*), started cannabis older than 17 (OR for 45 + = 5.1**)

^*^Statistically significant *p*-value < 0.05

**p-value < 0.01

^percent use compared M vs M + R using chi-square analysis

~Multinomial regression analysis

NS- not significant

### Source of cannabis & licensure of dispensaries

Frequency and demographic analysis for sources of cannabis are summarized in Table 5. The medicinal-only group predominantly sourced from dispensaries, whereas the M + R group utilized more varied sources for cannabis. Among all participants, most (94%) reported using licensed dispensaries (not shown in table). More participants from the medicinal-only group reported it being important or very important to use a licensed dispensary (72%) compared to the M + R group (66%, *p* < 0.01).

Among M + R users, those obtaining cannabis from family/friends were more likely to have had household income under $50 k, live in Northern California, and begun using cannabis before age 17 than those that did not get it from family/friends. M + R members who grew their own cannabis were more likely to be male, white, married, live in Northern California, and have started using cannabis at a younger age than those that did not grow their own cannabis. While both groups valued purchasing from licensed dispensaries, medicinal-only users reported this as very or extremely important at a significantly higher rate (72% vs 66%, *p* = 0.01) (Table 5).

### Positive and negative effects of cannabis use

Both medicinal groups reported positive effects of cannabis use, with the medicinal-only group seeing greater physical health benefits and the M + R group noting improvements in mental health, emotional health, thought clarity, focus, and maintaining healthy relationships (Table 6). After adjustment for other demographic variables, the M + R group members who reported a positive effect on their physical, mental and emotional health were more likely to be employed full-time. People from both groups who reported clarity and focus were more likely to be male and older.

**Table 6:** Positive and negative effects of cannabis among medicinal and M + R group

**Positive effect**	**Reasons for use**	**Percent use^**	**Demographical analysis & Odd ratio (comparing those who had positive effect to those who did not)**
Physical health	**M (*N*** **= 711)**	76%	Married (OR = 1.5*)
	**M + R (*N*** **= 1719)**	69%**	Employed full-time (vs unemployed, OR = 1.4**)
Mental health	**M**	76%	Started cannabis younger than 17 (OR for 45 + = 2.0**)
	**M + R**	88%**	Employed full-time (vs unemployed, OR = 1.6**)
Relationship	**M**	55%	Married (OR = 1.5**), Southern CA or central region (vs Northern, OR = 1.7–1.9**)
	**M + R**	62%**	Married (OR = 1.3*), employed full-time (vs unemployed, OR = 1.6**)
Emotional health	**M**	80%	Decreased with age
	**M + R**	88%**	Female (OR = 1.5*), employed full-time (vs unemployed, OR = 1.7**), Northern CA region (central, OR = 1.6*), use increased with age
Clear Head/Focus	**M**	59%	Male (OR = 1.4*), Hispanic or black (vs Asian/Pacific islander, OR = 2.4–4.3**), decreased with age**
	**M + R**	62%**	Male (OR = 1.4**), Hispanic (vs Asian/Pacific islander, OR = 1.5*), started cannabis younger than 17 (vs OR for 45 + = 1.7*), decreased with age
**Negative effect**	**Reasons for use**	**Percent use^**	**Demographical analysis & Odd ratio (comparing those who had negative effect to those who did not)**
Finances	**M**	17%	GED (vs college, OR = 1.8*), employed full-time (vs unemployed, OR = 2.1**), have kids in HH (vs no kids, OR = 2.2**), Central CA region (vs Northern, OR = 1.9*), decreased with age**
	**M + R**	21%**	Male (OR = 1.7**), employed full-time (vs unemployed, OR = 2.4**), have kids in HH (vs no kids, OR = 1.6**)
Paranoia	**M**	12%	Unemployed (vs employed full-time, OR = 2.2*), Northern CA region (vs Southern, OR = 2.2**), started cannabis younger than 17 (OR for 45 + = 4.8**)
	**M + R**	20%**	Started cannabis younger than 17 (OR for 45 + = 1.8*)
Fatigue	**M**	16%	Decreased with age**
	**M + R**	20%**	Decreased with age**
Brain fog	**M**	18%	–
	**M + R**	22%	Female (OR = 1.3*), single (OR = 1.3*)
Memory loss	**M**	10%	Started cannabis younger than 17 (OR for 45 + = 7.8**)
	**M + R**	17%**	Single (OR = 1.5**), started cannabis younger than 17 (OR for 45 + = 2.4*)
Lack of motivation	**M**	14%	Started cannabis younger than 17 (OR for 45 + = 2.7**)
	**M + R**	24%**	No kids in HH (vs have kids, OR = 1.4**), started cannabis younger than 17 (vs OR for 45 + = 1.7*)
Weight gain	**M**	14%	Started cannabis younger than 17 (OR for 45 + = 5.1**)
	**M + R**	20%**	No kids in HH (vs have kids, OR = 1.4**)
Dependency to cannabis	**M**	6%	Started cannabis younger than 17 (OR for 45 + = 6.6**)
	**M + R**	9%**	Male (OR = 1.4*), decreased with age

^*^Statistically significant *p*-value < 0.05

**p-value < 0.01

^percent use compared M vs M + R using chi-square analysis

~Multinomial regression analysis

The M + R group were also significant more likely than medicinal-only to report adverse effects, including finances (21% vs 17%, *p* < 0.001), paranoia (20 vs 12%, *p* < 0.001), fatigue (20 vs 16%, *p* < 0.001), memory loss (17 vs 10%, *p* < 0.001), lack of motivation (24 v 14%), weight gain (20 vs 14%, *p* < 0.001), and dependency (9 vs 6%, *p* < 0.001). Multivariate analysis is summarized in Table 7. Notably, many negative effects were associated with starting using cannabis at age 17 or younger.

**Table 7:** Pattern of cannabis use among medicinal and M + R group

Activity/Place	Reasons for use	Percent use^	Demographical analysis & Odd ratio (comparing stated activity/place vs not stated activity/place)
Use at home	**M (*N*** **= 711)**	93%	White non-Hispanic (OR = 3.5–4.3*), college & some college degree (vs graduate degree, OR = 4.2**)
	**M + R (*N*** **= 1719)**	94%	Female (OR = 1.8*), no kids in HH (vs kids, OR = 1.8*), started cannabis use < 17 (OR for 35–44 = 3.9**)
Someone else’s home	**M**	15%	GED (vs college degree, OR = 2.3**), employed part-time (vs unemployed, OR = 2.6**), started cannabis use < 17 (OR for 45 + = 6.2**), decreased with age**
	**M + R**	39%**	Single (OR = 1.4**), started cannabis use < 17 (OR for 45 + = 2.7**)
Parties/Indoor public	**M**	12%	Started cannabis use < 17 (vs OR for 45 + = 3.3**), decreased with age**
	**M + R**	38%**	Started cannabis use < 17 (vs OR for 45 + = 2.0**), graduate degree (vs GED, OR = 2.0**), decreased with age**
Outdoor public place	**M**	9%	Had kids in HH (vs no kids, OR = 2.0*), started cannabis use < 17 (OR for 45 + = 2.9**), decreased with age**
	**M + R**	27%**	Started cannabis use < 17 (vs OR for 45 + = 2.8**), decreased with age**
Work	**M**	5%	Have kids in HH (vs no kids, OR = 3.0*), graduate degree (vs college, OR = 3.2**), started cannabis use < 17 (OR for 45 + = 5.9**)
	**M + R**	10%**	Male (OR = 1.7**), not Asian/Pacific islander (OR = 0.4*), have kids in HH (vs no kids OR = 1.7**), decreased with age**
In car	**M**	2%	Started cannabis use < 17 (OR for 45 + = 5.9**), decreased with age**
	**M + R**	5%**	Started cannabis use < 17 (OR for 45 + = 2.6**), decreased with age**
Recreational creative activity	**M**	34%	White non-Hispanic (vs Hispanic, OR = 6.3*), started cannabis use < 17 (OR for 45 + = 12.3*)
	**M + R**	50%**	Male (OR = 1.9**), Northern CA region (vs Central, OR = 2.0**), started cannabis use < 17 (OR for 45 + = 3.4**), decreased age**
Drink alcohol	**M**	23%	Have kids in HH (vs no kids, OR = 5.3*)
	**M + R**	36%*	Male (OR = 1.3*), black non-Hispanic (vs Asian/pacific islander, OR = 1.7*)

*Statistically significant p-value 0.05

**p-value 0.01

^percent use compared M vs M+R using chi-square analysis

~Multinomial regression analysis

### Locations/activity during use

Most participants in both groups (93–94%) reported using cannabis at home, but the M + R group were more likely than the medicinal-only group to use in all other locations (see Table 7), during recreational creative activities (50 vs 34%, *p* < 0.001), and while drinking alcohol (36 vs 23%). Multivariate analysis is summarized in Table 7. Overall, starting using cannabis at age 17 or younger was associated with cannabis use outside the home in multiple categories. Those using cannabis at someone else's home were more likely to be single. Only 4% of participants in either group used cannabis when children were present (Not shown in table). For different product types, participants were asked how long they will wait before it feels safe to drive (Table 8). For all product types, medicinal-only users were significantly more likely to report waiting until the next day before they would feel safe to drive, while the M + R group was significantly more likely to report a time of less than 1 h before feeling safe to drive after cannabis use.

**Table 8:** Driving wait times by cannabis use type

**Time until feels safe to drive after flower use**	**Medicinal-only use N for flower = 239**	**Medicinal & Recreational Use (M + R)** ***N*** **= 1124**
An hour or less	19%	29%**
2–4 h	38%	32%
5 or more hours	1%	5%**
8 or more hours	4%	3%
Wait until next day	18%	10%**
**Time until feels safe to drive for edible use**	**Medicinal only** **N for edible = 399**	**M + R** ***N*** **= 984**
An hour or less	11%	16%*
2–4 h	20%	28%**
5 or more hours	5%	12%**
8 or more hours	8%	7%
Wait until next day	35%	24%**
**Time until feels safe to drive for vaping/dabbing**	**Medicinal only** **N for vape/dab = 208**	**M + R** ***N*** **= 907**
An hour or less	19%	27%**
2–4 h	25%	28%
5 or more hours	8%	7%
8 or more hours	8%	5%
Wait until next day	18%	12%*

^*^Statistically significant *p*-value < 0.05 compared medicinal-only vs M + R using chi-square analysis﻿

**p-value < 0.01

## Discussion

This study examined the profile of people who use medicinal cannabis in California, delving into usage patterns, cannabis sources, guidance sources, patient-provider relationships, and driving behavior. Sixty percent of current cannabis users in California reported medicinal use of cannabis, with or without concomitant recreational use. This increase aligns with evolving societal attitudes and the California Department of Health's observations of increased cannabis use since 2012 (Ryan-Ibarra et al. 2015; California Department of Public Health 2020). Compared to national data, in 2018, California's estimated cannabis use stands at 17.7% versus the national average of 10.5% (California Department of Public Health 2020). Earlier studies likely represent underreporting due to lower social acceptance, making our current insights critical for further research in this evolving landscape.

In this environment of increasing cannabis use, including for medicinal reasons, our study examined where Californian medicinal users look for guidance on their use. Clinicians and medical providers are not the primary sources regarding products and dosing; participants were more likely to seek guidance from sources like the internet, friends and family, and budtenders. This can be especially true of individuals with higher educational levels (who may feel in a position to better interpret online information) (Pew Research Center 2014) or are either Hispanic and/or Black non-Hispanic (who may have larger communities to share information with) (USAFacts 2025). To our knowledge, there are limited studies that describe the demographic analysis of those going to dispensaries, especially in California (Kaufmann et al. 2022; Rosenthal and Pipitone 2021). Instead, individuals may discuss use with budtenders, who come with more “lived” experience with cannabis, may bring personal biases, often have limited in-depth cannabis knowledge, and are also tasked with promoting sales (Slawek et al. 2023). For example, one study found that over 65% of surveyed budtenders recommended cannabis use during the first trimester of pregnancy, despite limited or concerning evidence regarding this practice (Merlin et al. 2021).

Despite reported concerns of fear of stigma from providers, the majority of participants viewed their primary care doctors and health groups as more accepting of cannabis and reported openly discussing their medicinal cannabis use with primary doctor. A 2022 qualitative survey showed that medicinal cannabis users perceive stigma from their providers, even when they feel comfortable disclosing use (Crapanzano et al. 2018). Yet, several studies of physicians have found in knowledge gaps, mixed attitudes, and inconsistent recommendations for medicinal cannabis use (Kruger et al. 2023; Adler et al. 2022). One health system study found that just 10% of physicians in USA had ever authorized medical cannabis, with low self-perceived knowledge and competency. Their knowledge is primarily focused on the risks of use, but providers feel ill-equipped to discuss aspects of medicinal use such as dosing and harm-reduction strategies. Medical school curricula do not equip providers with the knowledge and skills to discuss medicinal cannabis with patients (Zolotov et al. 2021). In addition, little guidance or support is provided by medical societies and physicians may fear losing their medical license due to its federal Schedule I status (Drug Enforcement Administration 2025; Rønne et al. 2021). Further research into providers' knowledge and attitudes regarding cannabis/cannabinoids, along with expanded training, would be an important future step; greater knowledge and awareness would empower providers to discuss medicinal cannabis use without bias, allowing them to address benefits, risks, and guidance effectively.

Participants in both groups primarily sought pain management, with the M + R group also emphasizing stress and anxiety relief. Despite limited evidence and particularly randomized clinical trials on cannabis benefits for mental health issues such as anxiety, depression, and attention deficit hyperactivity disorder (ADHD) (National Academies of Sciences, Engineering, and Medicine 2017; Francisco et al. 2023; Schlienz et al. 2021), both groups reported mental health benefits, including improved focus and mental clarity (Schlienz et al. 2021). Self-medication is likely, as about 60% in both groups of participants reported replacing prescription medications with cannabis. This is similar to the findings of 2022 survey, where participants cited multiple reasons for substituting cannabis for medication, including perceived treatment inefficacy, negative side effects from traditional pharmaceuticals or expressed a preference for a natural approach (Garcia-Romeu et al. 2022). Other studies have demonstrated that people view cannabis as a safer alternative to prescription opioids (Mattson et al. 2021). The M + R group reported more negative effects; this may be associated with the use of higher doses and higher potency products among recreational users (Urits et al. 2021), though that was not explored in this study. Given the known gaps in accessing mental health and substance use treatment, along with the opioid crisis, it is important that patients have access to safe cannabis use education, patient risk screening, and are supported medically so they do not resort to self-medication (Francisco et al. 2023; Mattson et al. 2021; Buttorff et al. 2023; Kohn et al. 2018).

This study additionally found that medicinal-only users were more likely to report occasional cannabis use (1–3 times per week or less), while the M + R group were more likely to report very frequent (multiple times a day) use. This differs from a 2023 study of recreational and medicinal cannabis users, which showed a similar frequency of use between groups, although problematic use was more common in recreational users (Ataiants et al. 2024). Medicinal-only users also reported a longer wait time before they felt safe to drive after using cannabis; future research should explore this difference, which could be associated with the demographics of medicinal-only users (e.g., female, older), or their use patterns (e.g., occasional use, use for sleep). While the decision to drive after cannabis use is multifactorial, recognizing the differences between medicinal-only and M + R users may help guide outreach toward the higher risk group.

There were some notable differences between groups about how cannabis is obtained and used. Our study found that about 60% of medicinal-only users obtained cannabis from dispensaries and delivery services, with fewer medicinal-only users acquiring it from friends and family than the M + R group; this is a similar finding to a post-Prop 64 survey of young adults in Los Angeles County (D’Amico et al. 2020). This also aligns with medicinal users prioritizing treatment over social or recreational use; dispensaries may be perceived as more reliable, regulated, and safer, and provide a higher variety of products to appeal to medicinal users (e.g., tinctures, salves). Although almost all participants in our study used cannabis at their own homes, the M + R group was more likely to also use cannabis in other, more public, locations. Similar to other studies (Subbaraman and Kerr 2018), the medicinal-only group also reported less concurrent alcohol use than the M + R group. Our study did not investigate cannabis product storage or child access (i.e., to what degree do users ensure restricted access to children), which is an important area for further research.

Medicinal-only users in our study were more likely to be female, consistent with previous findings (Jeffers et al. 2021). This pattern may reflect a greater tendency among females to seek medical care and a preference for more traditional and natural products (Bertakis et al. 2000). Medicinal-only users also initiated cannabis use later in life, possibly due to development of specific medical conditions. In contrast, combined M + R users were more likely to be male and initiate cannabis use at a younger age; these trends are in line with previous research comparing medicinal to recreational use (Goulet-Stock et al. 2017) and further demonstrates that medicinal use type should be considered. For example, studies have shown that early initiation of substance use during brain development is known to have associated risks including the risk for cannabis use disorder (CUD) (Urits et al. 2021; Narouze et al. 2024). Education and prevention efforts may be more effective when the target population’s medicinal cannabis use pattern is considered (Goulet-Stock et al. 2017).

This study’s strengths include the substantial sample size of 4,020 current users, closely matching California's demographic distribution. It incorporated algorithmic technology for efficient interaction in open-ended queries and benefited from guidance from SME interviews and the exploratory questionnaire. However, the study does have limitations. The study was confined to California, limiting its generalizability to states where cannabis use remains illegal, access to products is likely more limited, and attitudes towards use may differ. Since we were limited to querying individuals 21 years of age and older, the study does not provide insights into use by younger individuals. The online questionnaire required access to phones or the internet, potentially excluding marginalized groups, though the degree to which that limits participation has declined significantly over the past decade (Subbaraman and Kerr 2018).

## Conclusion

Cannabis use is growing with expanding legalization, necessitating more research to understand the ramifications of increased access, and better understand the factors influencing the choices and options available to users. Special attention should be given to medicinal users, who may represent a vulnerable group seeking symptom relief. Open discussions about medicinal use of cannabis can maximize potential benefits while limiting negative effects. Achieving this requires cooperation from various stakeholders, including patients, providers, government authorities, researchers, and industry.

## Data Availability

Data will be shared with approved researchers for meta-analysis upon request.

## Appendix 1

**Table:** Demographics of population by California census/target vs. demographics of unweighted/actual study population for screener questionnaire

	Unweighted	Target/weighted Screener (*n*=15,309)		Unweighted	Target/weighted Screener (15,309)
**Age**			**Region**		
21-34	28%	29%	**Northern Region**		
35-44	18%	19%	Superior California	8%	9%
45-54	17%	17%	North Coast	2%	2%
55+	37%	34%			
**Gender**			San Francisco Bay Area	18%	15%
Male	49%	48%			
Female	51%	52%	**Central region**		
**Ethnicity/Race**			Northern San Joaquin Valley	5%	4%
White non-Hispanic	40%	38%			
Black non-Hispanic	6%	6%	Central Coast	6%	5%
Hispanic (all races)	36%	37%	Southern San Joaquin Valley	7%	7%
Asian/Pacific Islander	16%	15%	**Southern region**		
Other	2%	2%	Inland Empire	12%	14%
**Annual Household Income**					
<$50k	26%	26%	Los Angeles	25%	27%
$50k - $99k	29%	33%	Orange	8%	8%
$100k	45%	41%	San Diego - Imperial	9%	9%

## Appendix 2

**Table:** Demographics of census-weighted/target current users vs. demographics of unweighted/actual current users

	Unweighted	Target/Weighted Current Users *N*=4,020		Unweighted	Target/Weighted Current Users *N*=4,020
**Age**			**Region**		
21-34	39%	41%	**Northern Region**		
35-44	22%	26%	Superior California	8%	9%
45-54	18%	16%	North Coast	2%	2%
55+	21%	17%			
**Gender**			San Francisco Bay Area	18%	15%
Male	59%	50%			
Female	41%	50%	**Central region**		
**Ethnicity/Race**			Northern San Joaquin Valley	5%	4%
White non-Hispanic	38%	39%			
Black non-Hispanic	8%	11%	Central Coast	6%	5%
Hispanic (all races)	42%	39%	Southern San Joaquin Valley	7%	7%
Asian/Pacific Islander	10%	9%	Southern region		
Other	2%	3%	Inland Empire	12%	11%
**Annual Household Income**					
<$50k	24%	34%	Los Angeles	25%	31%
$50k - $99k	28%	36%	Orange	8%	7%
$100k	48%	30%	San Diego - Imperial	9%	9%
